# Dromedary Milk Protein Hydrolysates Show Enhanced Antioxidant and Functional Properties

**DOI:** 10.17113/ftb.58.02.20.6337

**Published:** 2020-06

**Authors:** Olfa Oussaief, Zeineb Jrad, Isabelle Adt, Touhami Khorchani, Halima El-Hatmi

**Affiliations:** 1 Livestock and Wildlife Laboratory, Arid Lands Institute, University of Gabes, 4119 Medenine, Tunisia; 2 University of Lyon, University Claude Bernard Lyon 1, ISARA Lyon, BioDyMIA - Equipe Mixte d’Accueil n°3733, rue Henri de Boissieu, 01000 Bourg en Bresse, France; 3 Department of Food, High Institute of Applied Biology, University of Gabes, 4119 Medenine, Tunisia

**Keywords:** dromedary milk, proteolytic enzymes, protein hydrolysate, antioxidant activity, functional properties

## Abstract

**Research background:**

Milk protein hydrolysates have received particular attention due to their health-promoting effects. Dromedary milk differs from the milk of other dairy animals in the composition and structure of its protein components, which give it unique properties. The bioactivity and functionality of whole dromedary milk proteins and their enzymatic hydrolysates have not received much attention, hence this study aims to investigate the effect of enzymatic hydrolysis of dromedary milk proteins on their antioxidant activities and functional properties.

**Experimental approach:**

Dromedary milk proteins were treated using four proteolytic enzymes (pepsin, trypsin, α-chymotrypsin and papain) and two mixtures of enzymes (pancreatin and pronase). The degree of hydrolysis was measured to verify the hydrolysis of the proteins. The sodium dodecyl sulfate polyacrylamide gel electrophoresis (SDS-PAGE) and gel filtration chromatography served to determine the molecular mass distribution of the hydrolysates while reversed phase-high performance liquid chromatography (RP-HPLC) was conducted to explore their hydrophobicity. The antioxidant activities were evaluated using various *in vitro* tests, including 2,2-diphenyl-1-picrylhydrazyl (DPPH) and 2,2'-azino-bis(3-ethylbenzothiazoline-6-sulfonic acid **(**ABTS) radical scavenging capacities, iron(III) reducing ability and chelating activity. Besides, functional properties such as solubility, foaming and emulsification were assessed.

**Results and conclusions:**

Dromedary milk protein hydrolysates exhibited different degrees of hydrolysis ranging from 17.69 to 41.86%. Apart from that, the hydrolysates showed different electrophoretic patterns, molecular mass distribution and RP-HPLC profiles demonstrating the heterogeneity of the resulting peptides in terms of molecular mass and polarity. The hydrolysates displayed significantly higher antioxidant capacities than the undigested proteins at all the tested concentrations. Iron(II) chelating activity was the most improved assay after proteolysis and the hydrolysate generated with pancreatin had the highest chelating power. Dromedary milk protein hydrolysates possessed good solubility (>89%). Further, foaming and emulsifying properties of dromedary milk proteins were enhanced after their proteolysis. These interfacial properties were influenced by the enzymes employed during proteolysis.

**Novelty and scientific contribution:**

Enzymatic hydrolysis of dromedary milk proteins is an effective tool to obtain protein hydrolysates with great antioxidant and functional properties. These results suggest that dromedary milk protein hydrolysates could be used as a natural source of antioxidant peptides to formulate functional foods and nutraceuticals.

## INTRODUCTION

Endogenous generation of reactive oxygen species (ROS) is unavoidable in aerobic organisms because it is a consequence of normal metabolic processes. At normal levels, ROS are involved in mediating several cellular responses comprising cell growth and immunity (*1*). The production of ROS is exogenous sources such as exposure to air pollutants, radiation, pesticides and ozone. Thus, the excess of ROS leads to oxidative stress, which is related to the occurrence of numerous ailments like cancer, cardiovascular, inflammatory diseases and neurodegenerative disorders (*2*). Organisms possess antioxidant defence systems against oxidative stress like antioxidant thiols and enzymes. Nevertheless, under pathological or extreme environmental conditions, endogenous antioxidants are not sufficient to remove ROS and external sources of antioxidants are required (*3*). Moreover, ROS induce lipid peroxidation in foods which causes the reduction of their nutritive value and their shelf life (*4*). Hence, various synthetic antioxidants have been extensively utilized in pharmaceutical and food industries to prevent oxidative damage. However, the use of synthetic antioxidants is restricted due to their potential toxicity (*5*). Accordingly, there is an increasing interest in finding new and safe antioxidants from natural sources.

A large number of milk-derived peptides have been found to be a good source of natural antioxidants (*6*). Bioactive peptides could be released from milk proteins *via* enzymatic hydrolysis, which is the most efficient way to generate peptides with multiple biological activities like antioxidant, antimicrobial, anticancer and anti-inflammatory activities (*6*). Enzymatic proteolysis also improves protein functionality in terms of solubility, emulsification, and foaming ability. Therefore, protein hydrolysates have wide application in the food industry. They can be used as additives in beverages since they have excellent solubility. Besides, they could be used as foaming agents in ice cream, mousse and whipped toppings or as emulsifiers in salad dressings, meat products, cakes and so on (*7*, *8*).

Dromedary (*Camelus dromedarius*) milk differs from the milk of other dairy animals in the composition and structure of its proteins, which gives it different functional and bioactive properties. In fact, immunoglobulins, camel whey basic protein, peptidoglycan recognition protein and whey acidic protein are specific proteins found only in dromedary milk. Furthermore, dromedary milk is similar to human milk since it contains low amounts of κ-casein and high amounts of β-casein, lactoferrin and α-lactalbumin. It also lacks β-lactoglobulin, which makes it useful for those with cow’s milk allergy (*9*). Many studies have reported the medicinal properties of dromedary milk like anticancer, anti-diabetic and antihypertensive capacities as well as the ability to reduce autism symptoms (*10*, *11*). These therapeutic properties were attributed to its richness in vitamin C and minerals, its unique protein composition as well as its potential bioactive peptides released during the gastrointestinal digestion of milk proteins (*11*).

Functional and various bioactive properties of peptides obtained from cow’s milk proteins after enzymatic hydrolysis have been widely studied (*12*, *13*). Recently, bioactive peptides from dromedary milk have received great interest. The effect of enzymatic hydrolysis on the antioxidant activities of whey, casein or some individual proteins from dromedary milk has been reported (*14*, *15*). However, whole dromedary milk proteins and their hydrolysates have not received attention with respect to their bioactivity and functional properties. Therefore, this study investigates the antioxidant and functional properties of whole dromedary milk proteins before and after enzymatic hydrolysis by four proteolytic enzymes (pepsin, trypsin, α-chymotrypsin and papain) and two mixtures of enzymes (pancreatin and pronase).

## MATERIALS AND METHODS

### Materials

Dromedary milk was obtained from a dromedary (*Camelus dromedarius*) herd belonging to the Livestock and Wildlife laboratory, Arid Lands Institute of Medenine, Tunisia. Fresh dromedary milk was defatted by centrifugation (5000×*g,* 30 min, 4 °C, centrifuge Sorvall Lynx 6000; Thermo Fisher Scientific, Waltham, MA, USA). Then, it was lyophilized in a freeze dryer (Christ Gamma 1–20; Martin Christ GmbH, Osterode am Harz, Germany) and kept at –20 °C.

Pepsin from porcine stomach mucosa and pancreatin from porcine pancreas were obtained from Bio Basic (Ontario, Canada). Trypsin from porcine pancreas, α-chymotrypsin from bovine pancreas, papain from *Carica papaya* and pronase from *Streptomyces griseus* were purchased from Sigma-Aldrich, Merck (St. Louis, MO, USA). All other chemicals and reagents used were of analytical grade.

### Enzymatic hydrolysis of dromedary milk proteins

Dromedary milk protein hydrolysis was performed as described by Oussaief *et al*. (*16*) with slight modifications. Dromedary skimmed milk was resuspended on protein basis at 2.5% (*m/V*) in ultrapure water and the pH of this solution was adjusted to the optimal pH value of enzymes: 2 for pepsin, 8 for trypsin, α-chymotrypsin, pancreatin and pronase and 6.5 for papain, as given by the manufacturer. The hydrolysis was started by adding the enzymes to proteins at a ratio of 1:100 (by mass). The temperature of the reactions was maintained at 37 °C using a shaking water bath (LSB-030S; Daihan Labtech Co., Namyangju-si, Republic of Korea) at 150 rpm. After hydrolysis for 6 h, the enzymes were inactivated by heating the samples for 20 min at 85 °C. Dromedary milk protein hydrolysates (DMPHs) were neutralized to pH=7 and centrifuged at 10 000×*g* for 15 min at 4 °C. Then, the supernatants were freeze-dried and kept at −20 °C for further use. Control samples containing undigested dromedary milk proteins (UDMP) undergo the same procedure as the hydrolysates but without the addition of enzymes.

### Degree of hydrolysis

Degree of hydrolysis (DH) was measured as described by Hoyle and Merritt (*17*). A volume of 1 mL of each dromedary milk protein hydrolysate was added to 1 mL of a solution of 20% (*m/V*) trichloroacetic acid (TCA) and the mixtures were incubated at 25 °C for 30 min. Then, the mixtures were centrifuged at 10 000×*g* for 10 min at 4 °C to obtain 10% (*m/V*) TCA-soluble fraction. The total protein content of both 10% (*m/V*) TCA-soluble fractions and samples of dromedary milk protein hydrolysates were determined by the method of Lowry *et al*. (*18*). The DH value was calculated as the ratio of the 10% (*m/V*) TCA-soluble protein to the total protein in the sample, expressed as a percentage.

### Gel electrophoresis

Sodium dodecyl sulfate polyacrylamide gel electrophoresis (SDS-PAGE) was carried out according to the method of Laemmli and Favre (*19*) using a 5% (*m/V*) stacking gel and a 15% (*m/V*) separating gel. Samples were dissolved in a sample buffer (62.5 mM Tris-HCl buffer (pH=6.8), 2% (*m/V*) SDS, 10% (*V*/*V*) glycerol, 5% (*V*/*V*) β-mercaptoethanol and 0.0025% (*m/V*) bromophenol blue) at 1:1 (*V*/*V*) ratio and boiled for 3 min at 100 °C. Volumes of 10 μL of samples at 2 mg/mL proteins were loaded in the gel. After electrophoresis, proteins were fixed with 12% (*m/V*) TCA for 20 min, stained with 0.1% (*m/V*) Coomassie Brilliant Blue R-250 solubilized in a mixture of 50% (*V*/*V*) ethanol and 2% (*V*/*V*) TCA for 1 h and destained by several washes in 30% (*V*/*V*) ethanol, 10% (*V*/*V*) acetic acid solution.

### Gel filtration chromatography

Molecular mass distribution was determined using gel filtration chromatography by a Nexera XR HPLC system (Shimadzu, Tokyo, Japan) on a Superdex^®^ Peptide PE 7.5/300 column (GE Healthcare, Uppsala, Sweden) as described by Dupas *et al*. (*20*). A volume of 50 µL from each sample, filtered through a 0.45-μm syringe filters, was injected into the column. Elution was achieved at a flow rate of 0.25 mL/min with 30% (*V*/*V*) of 0.1% (*V*/*V*) trifluoroacetic acid (TFA) in acetonitrile and 70% (*V*/*V*) of 0.1% (*V*/*V*) TFA in water during 120 min. The elution was controlled spectrophotometrically at 215 nm (Cecil CE 2041; Cecil Instruments Ltd, Cambridge, UK). The column was previously calibrated using standard proteins: cytochrome C (12 400 Da), aprotonin (6500 Da), substance P (1348 Da), glycine 6 (360 Da), glycine 3 (189 Da) and glycine (75 Da).

### Reversed-phase high performance liquid chromatography

Reversed-phase high performance liquid chromatography (RP-HPLC) analysis was conducted using a Nexera XR HPLC system (Shimadzu) equipped with a C18 Omnispher column (250 mm×4.6 mm, 5 µm; GE Healthcare) as reported by Adt *et al*. (*21*). Samples were filtered through a 0.45-µm syringe filter (Millipore Corp., Billerica, MA, USA) and a volume of 50 µL of each filtered sample was loaded onto the column. Peptides were eluted with 0.1% (*V*/*V*) TFA in water for 10 min, followed by an 80-minute linear gradient from 0 to 50% (*V*/*V*) of acetonitrile in the presence of 0.1% TFA, then a linear gradient of 50 to 75% (*V*/*V*) acetonitrile in 0.1% (*V*/*V*) TFA during 10 min. The flow rate was 1 mL/min and the separation was monitored spectrophotometrically at 215 nm.

### Antioxidant properties

#### DPPH radical-scavenging activity

The scavenging activity of 2,2-diphenyl-1-piycrylhydrazyl (DPPH) radical was measured by the method of Bersuder *et al*. (*22*) with some modifications. A volume of 1 mL of each sample (1–7 mg/mL) was mixed with 1 mL of 125 µM DPPH in ethanol, kept in the dark for 60 min at room temperature and then the absorbance was measured at 517 nm using an UV-Vis spectrophotometer (Cecil CE 2041; Cecil Instruments Ltd). The control was prepared in the same way, except that distilled water was used instead of the sample. The DPPH radical-scavenging activity was calculated from the following equation: 


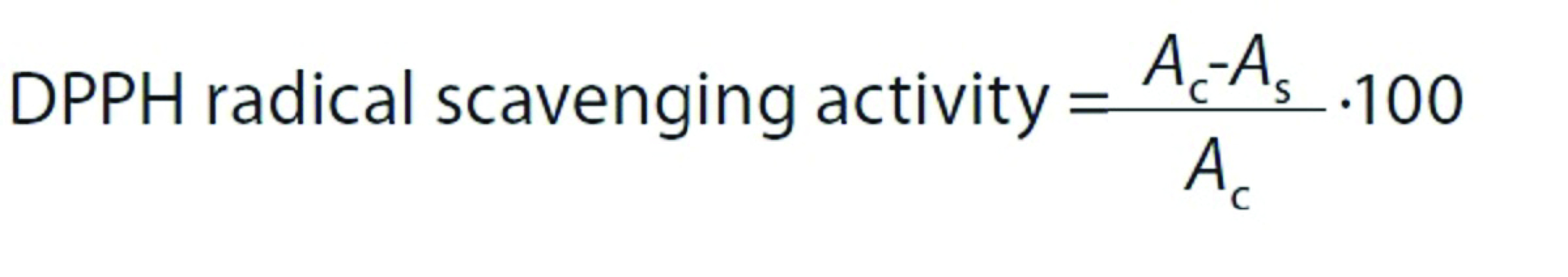


where *A*_c_ is the absorbance of the control and *A*_s_ is the absorbance of the sample.

#### ABTS radical-scavenging activity

The method of Re *et al*. (*23*) was used to determine the scavenging activity of the 2,2'-azino-bis(3-ethylbenzothiazoline-6-sulfonic acid (ABTS) radical, with minor modifications. The ABTS radical was generated by dissolving 7 mM of ABTS cation in 2.45 mM potassium peroxydisulfate and the mixture was kept in the dark at room temperature for 14 h. The ABTS radical cation solution was then diluted with sodium phosphate buffer (5 mM, pH=7.4) to obtain an absorbance of 0.7 at 734 nm. A volume of 0.25 mL of each sample with a concentration range from 0.25 to 1 mg/mL was added to 1 mL of the diluted ABTS radical reagent and left for 10 min at room temperature in dark conditions. Then, the absorbance was read at 734 nm. For the control, the distilled water was used instead of the sample. The ABTS scavenging effect was calculated using equation 1.

#### Ferric reducing antioxidant power

The reducing power of iron(III) ions was performed according to Wu *et al*. (*24*) with some modifications. A volume of 1 mL aliquot of each sample at different concentrations (1–20 mg/mL) was added to 1 mL phosphate buffer (0.2 M, pH=6.6) and 1 mL of 1% (*m/V*) of potassium hexacyanoferrate(III) and then heated for 20 min at 50 °C in a water bath. Then, 1 mL of 10% (*m/V*) TCA was added and the reaction mixtures were centrifuged for 10 min at 3000×*g*. Finally, a volume of 1 mL of the supernatant solution of each sample was added to 1 mL of distilled water and 0.2 mL of 0.1% (*m/V*) iron(III) chloride. After a 10-minute reaction, the absorbance of the resulting solution was measured at 700 nm. The control was prepared using distilled water instead of the sample.

#### Iron(II) chelating activity

The chelating ability of iron(II) ions was estimated as reported by Zhu *et al*. (*25*) with minor modifications. A volume of 1 mL aliquot from each sample solution (0.5–3 mg/mL) and 1 mL distilled water were added to 0.05 mL FeCl_2_ (2 mM). The mixtures were left for 30 s at room temperature. Then, a volume of 0.1 mL ferrozine solution (5 mM) was added to the reaction and the mixtures were incubated for 10 min at room temperature. The control was prepared in the same way, substituting the sample with distilled water. The absorbance was measured at 562 nm and the iron (II) chelating activity was calculated using the following formula:


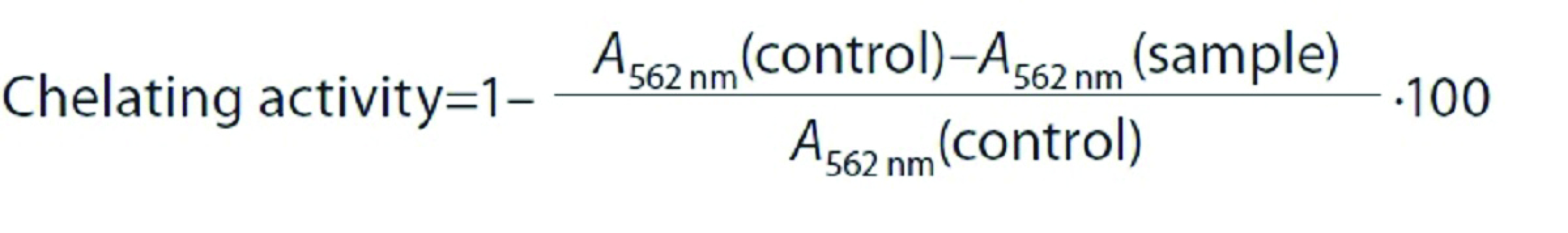


### Functional properties

#### Solubility

Solubility was estimated by the method of Tsumura *et al*. (*26*). Samples (100 mg) were dispersed in 10 mL of distilled water and the pH of the mixture was regulated to values from 3.0 to 9.0 with 2 M HCl or 2 M NaOH solutions. The mixtures were stirred at room temperature for 10 min and centrifuged at 8000×*g* for 10 min. The total protein content of the supernatants and samples of dromedary milk protein hydrolysates were determined by the method of Lowry *et al*. (*18*). Protein solubility was calculated as follows:


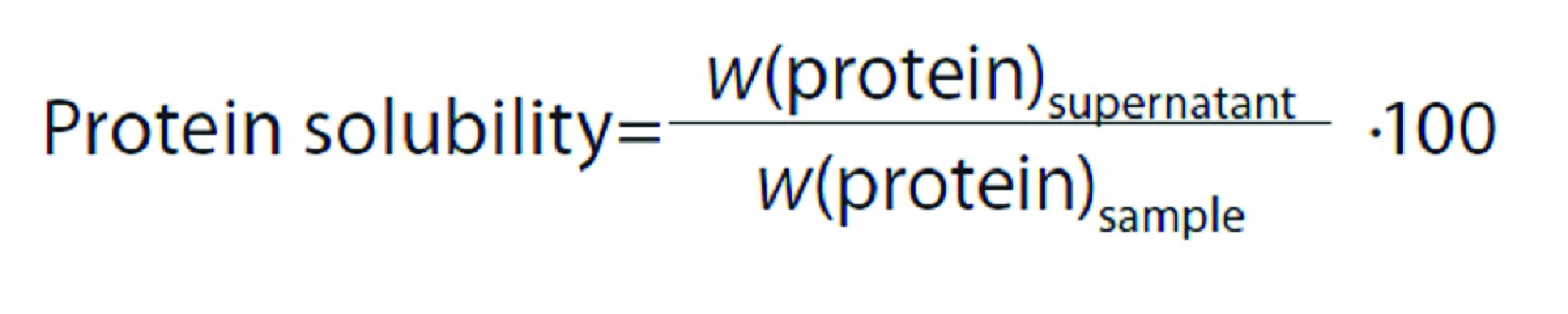


#### Foaming properties

Foaming properties were tested by the method of Shahidi *et al*. (*27*). A volume of 30 mL of 1% (*m/V*) sample was homogenized at room temperature using a blender (DOM216; DomoClip^®^, Mundelsheim, Germany) at the highest speed for 1 min, poured into a graduated cylinder and the total volume was recorded immediately. Foaming capacity was calculated according the following formula:


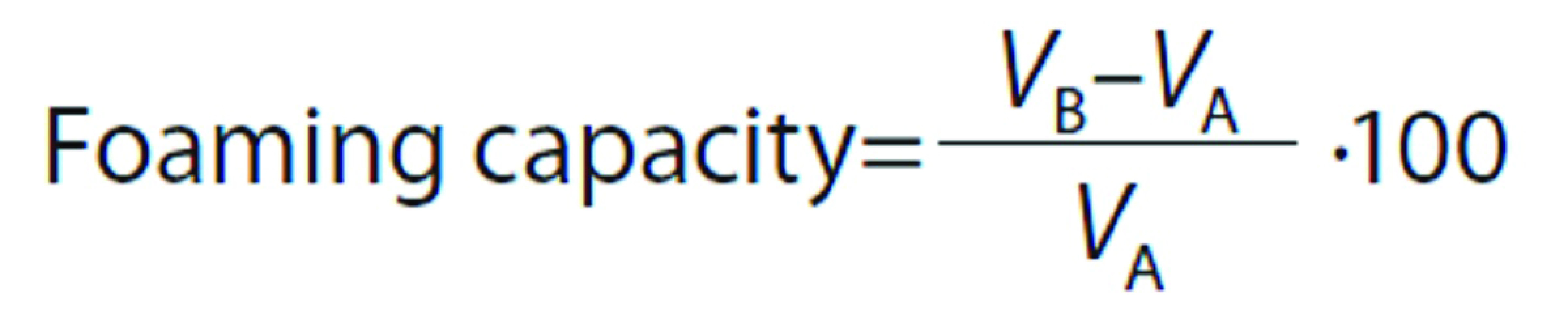


where *V*_B_ is the volume after whipping (mL) at 0 min and *V*_A_ is the volume before whipping (mL). After 30 min of standing at room temperature, the volume of whipped samples was recorded. Foam stability was calculated as follows:


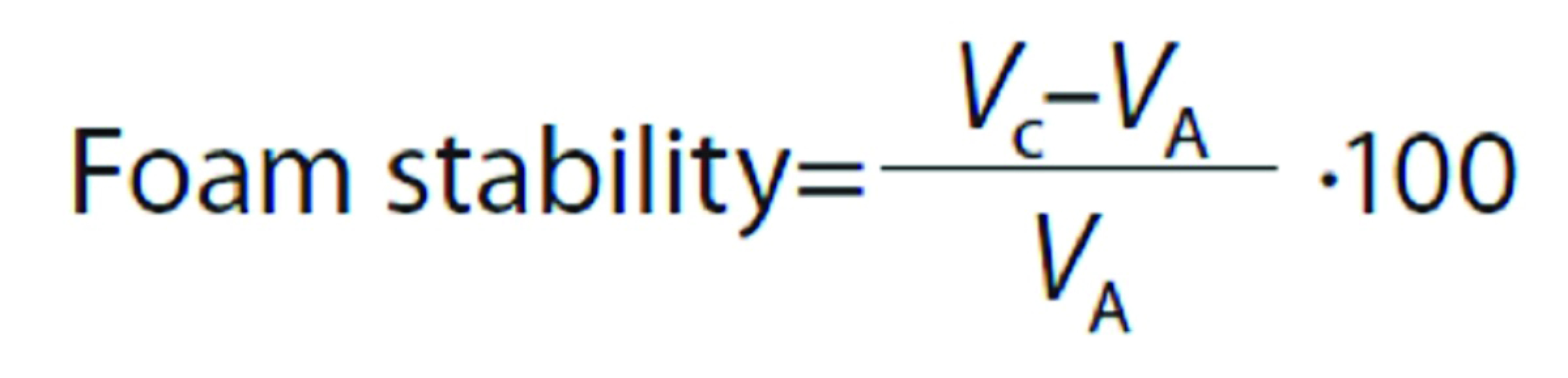


where *V*_A_ is the volume before whipping (mL), and *V*_C_ is the volume after 30 min of rest at room temperature (mL).

#### Emulsifying properties

The emulsifying activity index (EAI) and the emulsion stability index (ESI) were determined by the method of Pearce and Kinsella (*28*) with minor modifications. A volume of 30 mL of 1% (*m/V*) samples was homogenized at room temperature with 10 mL of corn oil for 1 min using a blender (DOM216; DomoClip^®^) at the highest speed. A 50-µL aliquot of the emulsion was taken from the bottom of the container at 0 and 10 min after homogenization and diluted 100 times with 0.1% (*m/V*) SDS solution. The absorbance of the diluted solutions was measured at 500 nm immediately (*A*_0_) and 10 min (*A*_10_) after the formation of the emulsion. The EAI and ESI were calculated as follows:


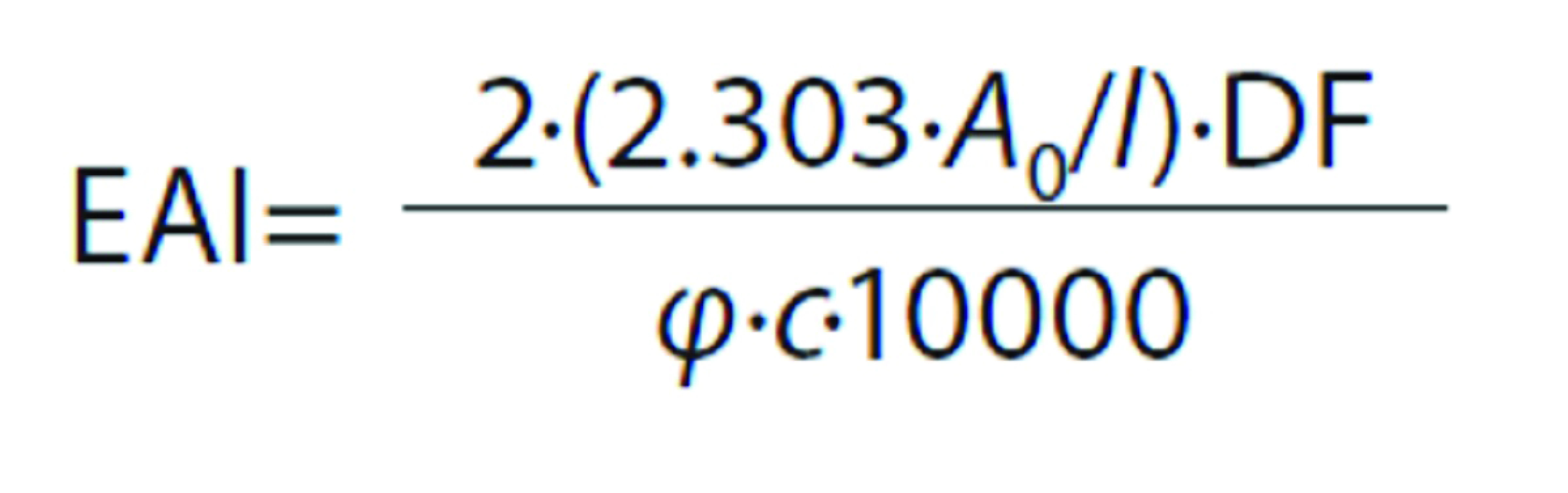


and


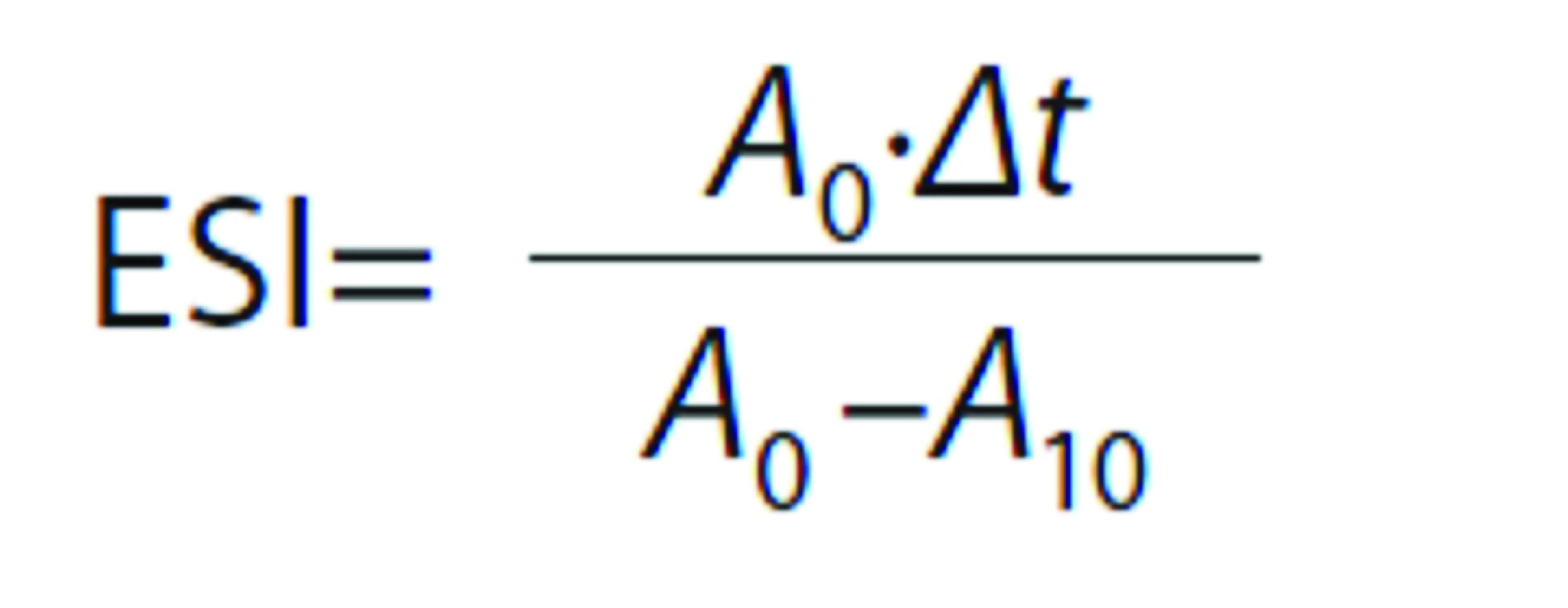


where *l* is cuvette length (1 cm), DF is dilution factor (100), *A*_0_ and *A*_10_ are the absorbance at time *t*=0 and 10 min, respectively, *c* is sample concentration (g/mL), and *φ* is the oil fraction (0.25).

### Statistical analysis

All experiments were conducted in triplicate. The results were statistically assessed using SPSS v. 22.0 (*29*). The one-way analysis of variance (ANOVA) was performed with Tukey’s test to determine the significance at 5% probability level.

## RESULTS AND DISCUSSION

### Preparation of dromedary milk protein hydrolysates

**Fig. 1:**
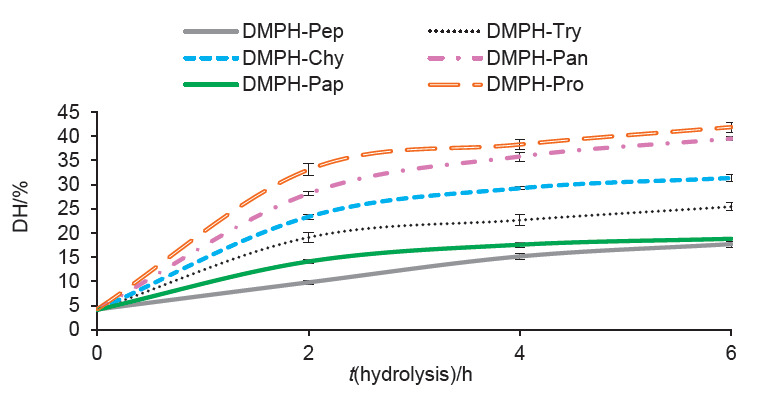
Degree of hydrolysis (DH) of dromedary milk proteins (DMPH) treated with pepsin (DMPH-Pep), trypsin (DMPH-Try), α-chymotrypsin (DMPH-Chy), pancreatin (DMPH-Pan), papain (DMPH-Pap) and pronase (DMPH-Pro)

In this study, various proteases were employed to hydrolyse dromedary milk proteins in order to assess the functionality of the generated protein hydrolysates. The extent of proteolysis in the hydrolysates was assessed by determining the degree of hydrolysis (DH). Fig. 1 shows the kinetic curves of dromedary milk proteins. During the first 2 h of hydrolysis, the DH increased rapidly, indicating that dromedary milk proteins contain many cleavage sites for the used enzymes. After that, the hydrolysis rate decreased, which might be a result of the reduction in available cleavage sites for the enzymes. The typical shape of hydrolysis curves was found previously for cow’s milk protein hydrolysates (*30*) and goat’s milk protein hydrolysates (*31*).

Pronase-treated hydrolysates showed the highest DH values, whereas pepsin-treated hydrolysates showed the lowest ones, at each time interval of hydrolysis. After 6 h of hydrolysis, DH values were 17.69, 25.41, 31.35, 39.48, 18.79 and 41.86% for DMPH treated with pepsin (Pep), trypsin (Try), α-chymotrypsin (Chy), pancreatin (Pan), papain (Pap) and pronase (Pro), respectively. Different DH values of milk proteins have been reported in the literature due to the variability of the specificity of the enzymes used and the amino acid sequence of proteins. Banach *et al*. (*32*) reported that the hydrolysis of cow’s milk proteins with pepsin for 12 h lead to a DH of 5.7%, whereas the hydrolysis of these proteins with papain for 3 h and trypsin for 1 h showed a DH values of 9.8 and 14.1%, respectively. The DH values of cow’s milk casein hydrolysates after 24 h of hydrolysis with trypsin, pancreatin and papain were 20.68, 20.91 and 22.06%, respectively (*33*). For dromedary casein hydrolysates, the DH was found to be 22 and 16% after 6 h of hydrolysis with α-chymotrypsin and papain, respectively (*15*).The DH values in the current study show that the used enzymes have a broad specificity on dromedary milk proteins compared with the above DH values.

The significant difference (p<0.05) in the DH among the hydrolysates of this study could be mainly due to the specificity of the used enzymes. Pronase has very broad specificity of action towards proteins since it contains several proteinases and peptidases from *Streptomyces griseus* (*34*). Pancreatin is a mixture of enzymes liberated by the pancreas and it also has a broad specificity, but it has a preference for Arg, Leu, Lys and Tyr (*35*). Papain cleaves the bonds of Lys, Arg and Phe. Chymotrypsin attacks at the carboxylic side of aromatic (Phe, Tyr and Trp) and long chain hydrophobic (Met and Leu) residues while trypsin splits peptide bonds in the carboxylic group of Arg and Lys residues. Besides, pepsin cleaves preferentially peptide bonds involving aromatic amino acids (*36*).

### Electrophoretic patterns of dromedary milk protein hydrolysates

**Fig. 2:**
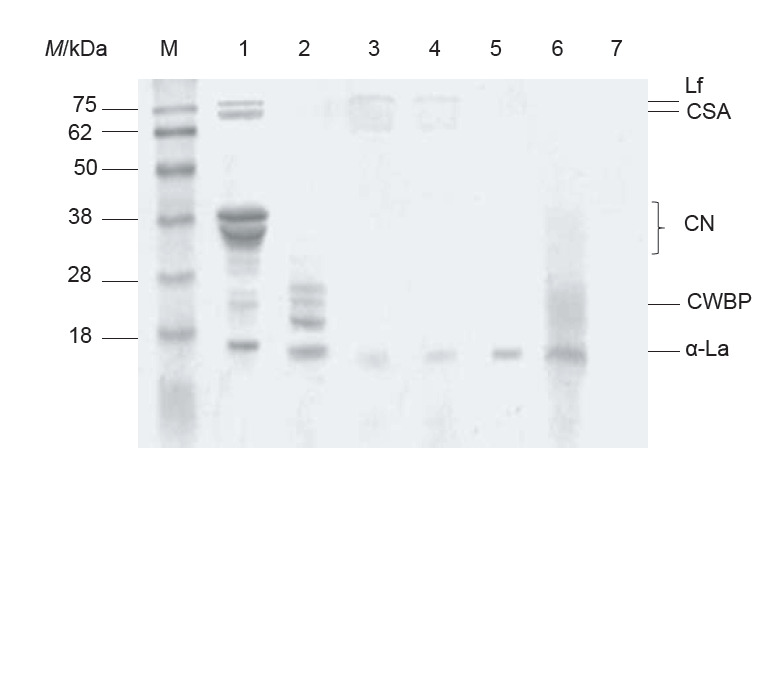
SDS-PAGE of undigested dromedary milk proteins (UDMP) and dromedary milk protein hydrolysates (DMPH) after 6 h of hydrolysis. Lane M=molecular mass marker, lane 1=UDMP, lane 2=DMPH-Pep, lane 3=DMPH-Try, lane 4=DMPH-Chy, lane 5=DMPH-Pan, lane 6=DMPH-Pap, lane 7=DMPH-Pro; Lf=lactoferrin, CSA=camelin serum albumin, CN=caseins, CWBP=camel whey basic protein and α-La=α-lactalbumin

The SDS-PAGE profiles of UDMP and DMPH are shown in Fig. 2. In this study, dromedary milk proteins before hydrolysis were predominantly lactoferrin, camel serum albumin, caseins, camel whey basic protein and α-lactalbumin. The protein profile of the hydrolysates changed. Indeed, lactoferrin band was absent from all the hydrolysates except from those obtained with trypsin and α-chymotrypsin, in which some traces of this protein were still noticed. In addition, bands of camel serum albumin and caseins were totally hydrolysed by all the enzymes into fragments invisible in the gel. Kumar *et al*. (*15*) also reported that dromedary milk caseins were rapidly hydrolysed with α-chymotrypsin and papain owing to their open structure. Camel whey base protein band was observed only in pepsin-treated hydrolysates. Except pronase, all the used enzymes exhibited limited degradation ability of α-lactalbumin. Banach *et al*. (*32*) reported that the hydrolysis of cow’s milk proteins with pepsin, trypsin and α-chymotrypsin degraded partially α-lactalbumin and β-lactoglobulin, whereas the rest of the proteins were all degraded into fragments invisible in the gel. The resistance of α-lactalbumin to proteolysis was attributed to its compact globular structure which hides its cleavage sites (*14*). Overall, most of high-molecular-mass proteins were degraded after enzymatic hydrolysis. The differences in enzyme specificity towards proteins might cause variability in protein degradation. These results suggest that the highest proteolysis occurred with pronase and the lowest with pepsin, which is in agreement with the results obtained for DH.

### Molecular mass distribution of dromedary milk protein hydrolysates

Gel filtration chromatography was performed to determine the molecular mass distribution of peptides in each sample (Table 1). UDMP had the lowest amount of low molecular mass peptides below 1 kDa (23.15%). After hydrolysis, the level of peptides with molecular mass higher than 10 kDa decreased, while the level of low molecular mass peptides (<1 kDa) increased, which confirms the generation of shorter peptides during the enzymatic hydrolysis. The differences in molecular mass peptide distribution depended on the used enzyme. Besides, the content of low molecular mass peptides positively correlated with the DH of the hydrolysates. In fact, hydrolysate DMPH-Pro, with the highest DH, comprised the highest amount of peptides with molecular mass under 1 kDa (86.41%), followed by DMPH-Pan (82.02%). For the other hydrolysates, the amount of low molecular mass peptides below 1 kDa ranged from 46.22 to 68.38%. Amiot *et al*. (*37*) reported that the hydrolysis of cow’s milk protein using trypsin (DH=6%) resulted in hydrolysates containing 60% peptides with molecular mass under 3 kDa. In addition, these authors found that cow’s milk protein hydrolysates obtained with α-chymotrypsin (DH=6%) contained 80% peptides with molecular mass below 1.2 kDa.

**Table 1:** Molecular mass distribution (%) of undigested dromedary milk proteins (UDMP) and dromedary milk protein hydrolysates (DMPHs)

	*M*/kDa			
Sample	>10	10–5	5–1	<1
UDMP	68.31**^a^**	0.00**^f^**	8.54**^e^**	23.15**^g^**
DMPH-Pep	25.67**^b^**	1.87**^c^**	26.23**^b^**	46.22**^f^**
DMPH-Try	16.19**^e^**	6.48**^a^**	22.13**^c^**	55.20**^d^**
DMPH-Chy	18.06**^c^**	2.22**^b^**	11.35**^d^**	68.38**^c^**
DMPH-Pan	14.05**^f^**	1.01**^e^**	2.86**^g^**	82.08**^b^**
DMPH-Pap	16.62**^d^**	1.07**^d^**	30.00**^a^**	52.31**^e^**
DMPH-Pro	8.60**^g^**	0.00**^f^**	4.99**^f^**	86.41**^a^**

DMPHs were obtained by hydrolysis of dromedary milk proteins by pepsin (DMPH-Pep), trypsin (DMPH-Try), α-chymotrypsin (DMPH-Chy), pancreatin (DMPH-Pan), papain (DMPH-Pap) and pronase (DMPH-Pro)

^a-g^Different letters in the same column indicate significant differences (p<0.05)

### Analysis of dromedary milk protein hydrolysates by RP-HPLC

**Fig. 3:**
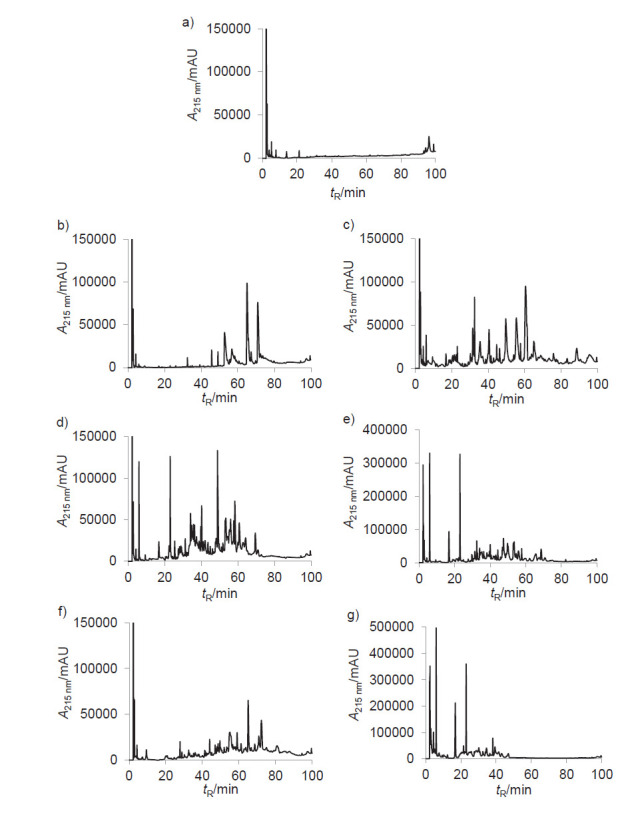
Reversed phase-HPLC profiles of undigested dromedary milk proteins (UDMP) and their hydrolysates (DMPHs): a) UDMP, with b) pepsin, c) trypsin, d) α-chymotrypsin, e) pancreatin, f) papain and g) pronase

RP-HPLC is a good method to investigate the hydrophobicity of proteins and peptides (*21*). The RP-HPLC elution profiles of UDMP and DMPH are visible in Fig. 3. A few peaks with low intensities were detected in the chromatogram of the UDMP, which indicated that they contained only a few peptides. After hydrolysis, numerous peaks were eluted between 20 and 80 min in the DMPHs, confirming the hydrolysis of dromedary milk proteins into several peptides. These results are in agreement with the DH measurements, SDS-PAGE patterns and molecular mass distribution. During proteolysis, the breakdown of every peptide bond releases two highly hydrophilic chemical groups. Subsequently, the hydrophobicity as well as the molecular mass distribution of the hydrolysates decreased compared to the native proteins (*38*). The hydrolysates comprised peptides with different hydrophobic and/or hydrophilic properties. In fact, both pepsin- and papain-treated hydrolysates showed the highest content of hydrophobic (high retention time) peptides. However, pronase-treated hydrolysates, with the highest DH, contained more hydrophilic (low retention time) peptides than the other hydrolysates.

### Effects of enzymatic hydrolysis on the antioxidant activities of dromedary milk proteins

**Fig. 4:**
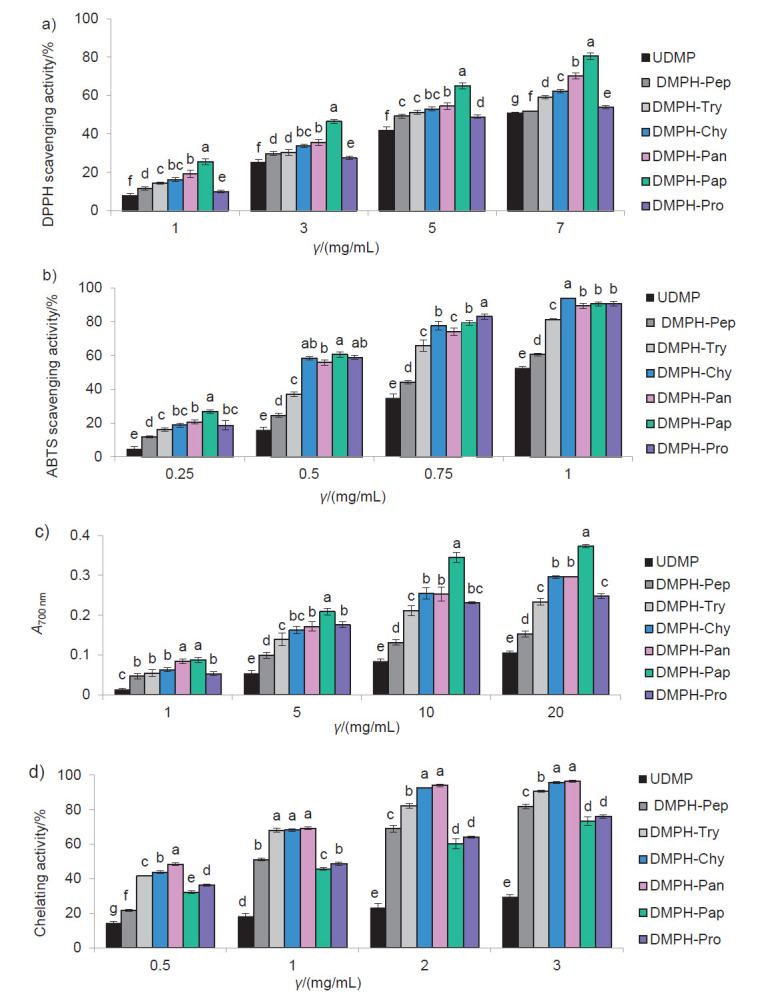
Antioxidant activities of undigested dromedary milk proteins (UDMP) and their hydrolysates (DMPHs) as a function of their concentrations: a) DPPH radical-scavenging activity, b) ABTS radical-scavenging activity, c) FRAP (ferric reducing power assay), and d) iron(II) chelating activity. All results are expressed as mean value±S.D. of triplicate measurements; S.D.=standard deviation. Values with different lowercase letters at the same concentration are significantly different (p<0.05)

Fig. 4 a reports the DPPH radical-scavenging capacity of UDMP and DMPHs, at various concentrations. Both UDMP and DMPH were able to scavenge DPPH radical in a concentration-dependent manner. DMPH possessed greater DPPH scavenging capacity than UDMP (p<0.05). These results demonstrate that enzymatic hydrolysis of dromedary milk proteins leads to the liberation of peptides able to scavenge the DPPH radical (*39*). Whatever the concentration tested, DMPH-Pap exhibited the greatest DPPH scavenging activity (p<0.05). IC_50_, defined as the concentration of samples required to inhibit 50% of the initial DPPH radical activity, was the lowest in DMPH-Pap (3.47 mg/mL). IC_50_ values were between 4.43 and 5.23 mg/mL for the rest of the hydrolysates. DMPH had higher DPPH scavenging activity than dromedary casein hydrolysates determined by Kumar *et al.* (*15*), who found that the DPPH scavenging activity did not exceed 40%. These findings confirm that DPPH scavenging activity of DMPH resulted not only from the proteolysis of casein but also from the proteolysis of whey proteins. Therefore, proteolysis of whole dromedary proteins provides greater DPPH scavenging activity than casein and whey hydrolysed independently.

Fig. 4 b shows the results of ABTS scavenging activity of UDMP and DMPH. All the samples had the ability to quench the ABTS also in a concentration-dependent manner. ABTS scavenging capacity was more pronounced in DMPH than in the UDMP (p<0.05). These findings are similar to those by Kumar *et al*. (*15*), indicating that dromedary casein hydrolysates had higher ABTS scavenging activity than intact dromedary caseins. DMPH-Pap had the lowest IC_50_ value (0.417 mg/mL), while UDMP had the highest (0.967 mg/mL). The IC_50_ values for the other hydrolysates were between 0.437 and 0.836 mg/mL. These results are in agreement with the hydrolysates from dromedary colostrum proteins (*16*). However, Oh *et al*. (*40*) reported higher IC_50_ values for cow’s milk protein hydrolysates than those in the present study. These findings may be explained by the fact that dromedary milk possesses higher content of antioxidant proteins like α-lactalbumin and β-casein than cow’s milk (*9*, *14*).

Both UDMP and DMPH scavenged more ABTS radicals than DPPH radicals. This difference could be due to the capacity of ABTS and DPPH radicals to diffuse in the reaction medium. ABTS is soluble in alcoholic and aqueous solutions and then it could easily react with peptides present in an aqueous medium. However, DPPH is soluble only in alcoholic solutions and may not reach readily the peptides in aqueous media. Subsequently, a high capacity to scavenge ABTS does not always implicate a high capacity to quench DPPH (*41*).

Fig. 4 c presents the ferric reducing antioxidant power (FRAP) of UDMP and DMPH at various concentrations. The FRAP of all the samples was concentration dependent; it increased when the concentration of the samples increased. At all the tested concentrations, UDMP exhibited lower reducing power than DMPH (p<0.05). The improvement of the FRAP of dromedary milk proteins after enzymatic hydrolysis might be due to the increased availability of peptides able to reduce iron(III) ions. At a concentration range from 5 to 20 mg/mL, DMPH-Pap, which exhibited the highest DPPH scavenging ability, had also the highest FRAP, while DMPH-Pep had the lowest FRAP. The difference in the FRAP among the hydrolysates might be assigned to the specificity of the used enzymes. Similar results were registered for dromedary and cow’s milk casein hydrolysates (*15*, *33*).

Fig. 4 d illustrates the chelating activity of UDMP and DMPH. The chelating power of Fe^2+^ increased considerably after the enzymatic hydrolysis of dromedary milk proteins. This observation could be due to the formation of amino acids with high metal-binding capacity (*42*). The hydrolysates chelated iron(II) ions in a concentration-dependent manner. The iron(II) chelating activity of the hydrolysates obtained from cow’s milk proteins (1 mg/mL) prepared by Conway *et al*. (*43*) did not exceed 25.5%, while for DMPH (at the same mass concentration) it was in the range of 45.67-69.20%. Indeed, dromedary milk contains higher amounts of lactoferrin than cow’s milk, and this protein is known as a good iron chelator (*11*). Among the different hydrolysates, DMPH-Pan exhibited the lowest IC_50_ value (0.54 mg/mL), followed by DMPH-Chy (0.61 mg/mL), while the highest one was obtained with DMPH-Pap (1.35 mg/mL) (p<0.05). The difference in the chelating capacity among hydrolysates could be due to the difference in their amino acid sequence and composition of peptides since they were generated using enzymes with different specificities.

### Effects of enzymatic hydrolysis on the functional properties of dromedary milk proteins

**Fig. 5:**
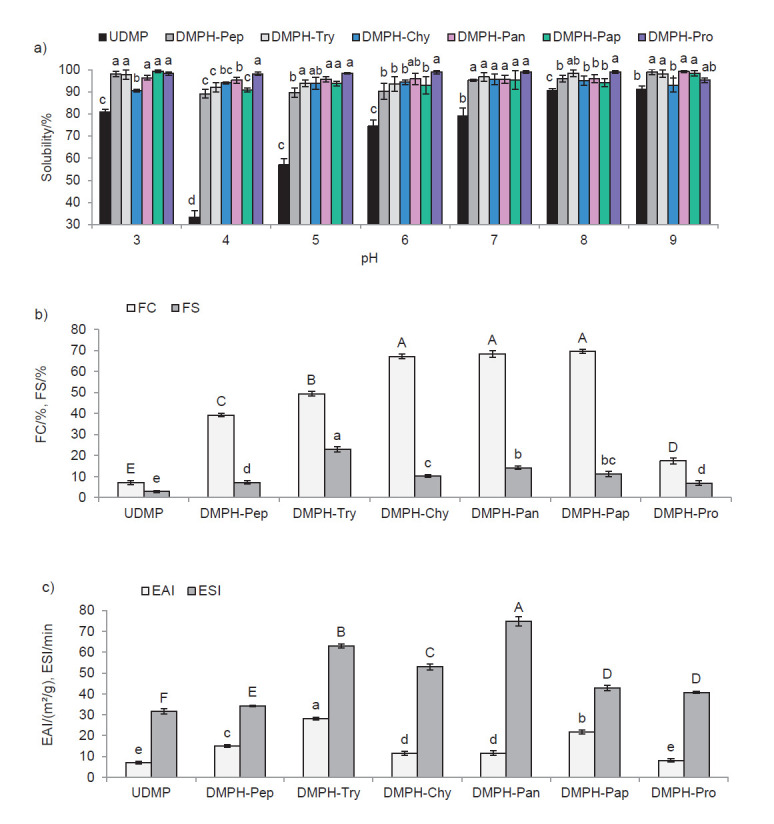
Functional properties of undigested dromedary milk proteins (UDMP) and dromedary milk protein hydrolysates (DMPHs): a) solubility at the same pH. Values with different lowercase letters are significantly different (p<0.05), b) foaming properties. Values with different lowercase letters indicate significant differences in foam stability and values with uppercase letters indicate significant differences in foaming capacity (p<0.05), and c) emulsifying properties. Values with different lowercase letters indicate significant differences in the emulsifying activity index (EAI) and values with uppercase letters indicate significant differences in the emulsion stability index (ESI) (p<0.05). All results are mean value±S.D. of triplicate measurements

Solubility of proteins is a significant attribute, which is required for use in many functional applications because it influences other properties like foaming and emulsifying capacities. Solubility of UDMP and DMPHs at different pH values is shown in Fig. 5 a. Interestingly, enzymatic hydrolysis enhanced the solubility of dromedary milk proteins. Solubility of UDMP was minimum at pH=4.0 (33.11%), which is near the isoelectric point of dromedary milk caseins, and increased below and above this pH value. However, solubility of DMPHs exceeded 89% over the entire measured pH range. DMPH-Pro, with the highest DH (41.86%) and lowest molecular mass peptides, exhibited a high solubility (>98%) at pH=4.0 to 8.0. The improvement of the solubility of protein hydrolysates could be explained by the reduction of the molecular mass of proteins and the increase of ionizable groups (amino and carboxyl groups), which could enable the protein to build hydrogen bonds with water (*38*).

The foaming properties of proteins depend on their capacity to migrate to the air-water interface in order to decrease the surface tension (*44*). Foaming capacity and foam stability of DMPH and UDMP are shown in Fig. 5 b. The hydrolysates had higher foaming capacity than UDMP (p<0.05). The highest foaming capacity was found in DMPH-Chy, DMPH-Pan and DMPH-Pap and it was 67.11, 68.33 and 69.44% respectively, whereas UDMP had the lowest foaming capacity (7.22%). Gani *et al*. (*13*) also indicated that enzymatic hydrolysis of cow’s milk proteins increased foaming capacity. Its enhancement after enzymatic proteolysis could be due to the generation of amphiphilic peptides which could migrate more rapidly to the air–water interface to encapsulate air particles. Foaming expansion after whipping was monitored for 30 min to study the foam stability. DMPH-Try showed the highest foam stability (23.06%). The foam stability decreased with time (p<0.05), which is explained by the fact that some peptides did not have the capacity to maintain stable foam (*13*).

Emulsifying properties of DMPH and UDMP reported in terms of EAI and ESI are observable in Fig. 5 c. The EAI indicates the capacity of a protein to form an emulsion, while ESI reflects the ability of an emulsion to maintain unvarying properties for a certain period (*45*). EAI were significantly higher (p<0.05) in the hydrolysates than in the UDMP. The highest EAI was obtained with DMPH-Try (28.18 m^2^/g). This could be due to the presence of high amounts of amphiphilic peptides in this fraction that possess a great flexibility at the oil/water interface, which results in a large surface area, and, enhance the formation of an emulsion. However, the lowest EAI value was found in DMPH-Pro, the hydrolysate with the highest DH (8.05 m^2^/g). This observation could be explained by the existence of small peptides, which are less efficient in emulsion stabilization (*46*). The ESI of the hydrolysates was in the range of 34.28–74.82 min and the highest value was found in DMPH-Pan (p<0.05). Enzymatic hydrolysis generates peptides, which are surface active owing to their hydrophobic and hydrophilic groups. Thus, the EAI and ESI of the hydrolysates were enhanced. Emulsifying properties of the hydrolysates were influenced by the properties of the generated peptides including size, amphiphilicity and flexibility (*46*). In the same context, improvement of the emulsifying properties of cow’s milk protein hydrolysates was found by Luo *et al*. (*33*).

## CONCLUSIONS

In this work, different hydrolysates were prepared from dromedary milk proteins (DMPH) by single enzymes as well as a mixture of proteolytic enzymes. The degree of hydrolysis, SDS-PAGE, RP-HPLC and molecular mass distribution were studied to characterize the generated protein hydrolysates. DMPH exhibited significantly higher antioxidant activities, in various *in vitro* assays, than the undigested proteins. In addition, enzymatic hydrolysis of dromedary milk proteins enhanced the solubility, foaming and emulsifying properties. The differences in the antioxidant activities and functional properties among the hydrolysates could be due to the used enzymes since they have different specificities. Therefore, such DMPHs could be used as natural antioxidant ingredients in functional food formulations. Further investigations are needed to purify and identify potent antioxidant peptides from DMPHs and test them *in vivo*.
